# Fibroblast growth factor 10 protects against UVB‐induced skin injury by activating the ERK/YAP signalling pathway

**DOI:** 10.1111/cpr.13315

**Published:** 2022-07-18

**Authors:** Nan Wang, Yetong Dong, Xiejun Xu, Yingjie Shen, Zhiyuan Huang, Yin Yu, Zhili Liu, Wenjie Gong, Siyi Zhang, Yeyi Zheng, Yonghuan Song, Zhongxin Zhu, Litai Jin, Weitao Cong

**Affiliations:** ^1^ School of Pharmaceutical Science Wenzhou Medical University Wenzhou People's Republic of China; ^2^ College of Pharmacy and Research Institute for Drug Development Chonnam National University Gwangju Republic of Korea; ^3^ Department of Orthopaedics The Second Affiliated Hospital and Yuying Children's Hospital of Wenzhou Medical University Wenzhou People's Republic of China

## Abstract

**Objectives:**

Ultraviolet light B (UVB) irradiation can induce skin injury and result in keratinocytes proliferation inhibition. However, the molecular understanding of the repair during UVB‐induced cell proliferation inhibition remains poorly understood. The purpose of this study was to explore the role and potential mechanism of FGF10 in promoting keratinocytes cell cycle and proliferation after UVB injury.

**Materials and Methods:**

Expression of FGF10 protein was analysed in skin treated with UVB radiation by immunohistochemistry. The proliferation potential was examined by Immunofluorescence, Western Blot and RT‐PCR under UVB radiation, treated with FGF10 protein or overexpression of FGF10 using adeno‐associated virus. CCK8 kit was used to further detect cell proliferation ability.

**Results:**

We found that FGF10 is highly expressed in skin treated with UVB. Overexpression of FGF10 has a protective effect against UVB‐induced skin damage by balancing epidermal thickness and enhancing epidermal keratinocytes proliferation. Importantly, FGF10 is found to alleviate UVB‐induced downregulation of YAP activity, then promoting keratinocytes proliferation. Disruption of YAP function, either with the small molecule YAP inhibitor Verteporfin (VP) or YAP small‐interfering RNA (siRNA), largely abolishes the protective activity of FGF10 on epidermal keratinocytes proliferation. Meanwhile, disruption of ERK kinase (MEK) activity with U0126 or ERK siRNA hinder the positive influence of FGF10 on UVB‐induced skin injury.

**Conclusion:**

FGF10 promotes epidermal keratinocytes proliferation during UVB‐induced skin injury in an ERK/YAP‐dependent manner.

## Graphical abstract

Schematic illustration of the protective effects of FGF10 on keratinocytes under UVB radiation. UVB radiation would cause skin injury and proliferation inhibition. In this study, we revealed that FGF10 promotes keratinocytes cell cycle and proliferation via ERK/YAP signalling pathway.

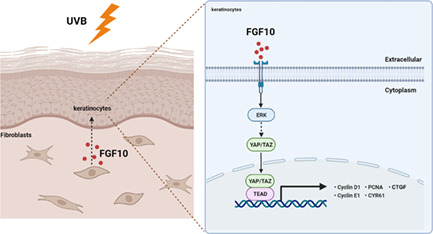

## 1 INTRODUCTION

Skin is the largest organ in our body and primarily serves as a protective barrier against the external environment.^1^, ^2^ It can effectively prevent the invasion of harmful substances and pathogens, maintaining the stability of the internal environment.^3^

UVB (280–320 nm) radiation, one of the most damaging solar UV emissions, can affect various skin structures, causing edema, erythema, hyperplasia, wrinkling, roughness, and premature aging, and can lead to the cell cycle and proliferation inihibition.^4^, ^5^ Studies have revealed that chronic exposure of skin to UVB irradiation increases the level of reactive oxygen species (ROS), resulting in oxidative damage to lipids, proteins, and nucleic acids within cells, leading to inflammation, immunosuppression, apoptosis and mutation.^6^

Fibroblast growth factors (FGFs) are a family of cytokine that play an important role in growth, development, metabolism and disease.^7^, ^8^ Phylogenetic analysis of the *Fgf* gene family identifies seven subfamilies, indicating potential evolutionary relationships between members of this gene family.^9^, ^10^ Fibroblast growth factor 10 (FGF10), a member of the FGF7 subfamily, is secreted by mesenchymal cells such as fibroblasts, endothelial, and inflammatory cells, and specifically binds to FGFR2 IIIb on epithelial cells to regulate embryogenesis and adult tissue homeostasis.^11^ It has also been reported that FGF10 can play diverse roles in epithelial‐mesenchymal transition, the repair of tissue injury and embryonic stem cell differentiation. Furthermore, FGF10 plays essential roles in proliferation, differentiation, migration and movement of epithelial cells via mechanisms closely related to those required for organ development.^12^, ^13^ FGF10 is an important growth factor for keratinocytes, stimulating keratinocyte proliferation, and participates in the cell cycle, DNA repair, apoptosis, development and wound healing.^12^ However, the role of FGF10 plays during UVB‐induced skin injury is still unknown.

In mammals, Yes‐associated protein (YAP) is a core component of the Hippo signalling pathway, a network of proteins that regulate body growth.^14^, ^15^ When the Hippo pathway is turned off, YAP and TAZ become dephosphorylated and translocate to the nucleus, where they are able to activate target genes that regulate cell growth, metabolism, proliferation, migration, invasion and cell death.^16^ Additionally, a number of studies have reported that YAP participates in the regulation of tissue regeneration, and plays an important role in epidermal healing after trauma.^17^, ^18^, ^19^ There is also growing evidences suggest that YAP takes part in signalling pathways that promote epithelial‐mesenchymal transition, enhance the ability of epidermal cells to migrate, and accelerate wound healing.^20^, ^21^ Although YAP has been shown to play a role in cell growth, little is known about its role in the response to injury to the skin epidermis caused by UVB.

In the present study, we show that FGF10 protects against UVB‐induced skin injury by alleviating DNA damage, balancing epidermal thickness, and promoting keratinocyte proliferation. We found that these protective effects are largely dependent on ERK‐mediated YAP activation. The data presented here unveils a new role for FGF10 in the regulation of UVB‐induced skin injury.

## 2 MATERIALS AND METHODS

### 2.1 Animals

Male C57BL/6 mice at the age of 8‐ to 10‐week‐old were used in this study. Mice were housed in temperature‐controlled pathogen‐free facility with 12‐h light/dark cycle and had access to food and water ad libitum. All animal procedures were approved by the Institutional Animal Care and Use Committee of Wenzhou Medical University.

For studies in FGF10 overexpression, each C57BL/6 mice was subcutaneously injected with a single dose of 1 × 10^11^ vector genomes of adeno‐associated virus (AAV) serotype 9 carrying either Fgf10 (AAV‐*Fgf10*) or GFP control gene (AAV‐*GFP*) with the CMV promoter (All of which contain the GFP tag). For mice virus transfection, 3 weeks later after the injection, mice were started to use for further experiments. AAV‐*GFP*, AAV‐*Fgf10* (contract number: HYSW‐BD‐PTHC‐2018100004) were constructed and purchased from OBiO Technology (Shanghai) Corp., Ltd.

For in vivo YAP/TEAD interact inhibition, mice were subcutaneous injected with saline or Verteporfin (20 mg/kg; Selleck) 1 h prior to UVB radiation. While for the ERK activity inhibition, mouse was subcutaneous injected with saline or U0126 (5 mg/kg; Selleck) 1 h prior to UVB radiation.

### 2.2 UVB‐irradiated animal experiments

Mice were exposed to UVB 2 weeks with a total energy dose of 200 mJ/cm^2^ and were sacrificed after 24 h of the last UVB exposure. Histopathology examination and immunohistochemical analysis were performed by placing a part of the dorsal skin in 10% phosphate‐buffered formalin. The remainder of the skin tissues was stored in liquid nitrogen.

### 2.3 Cell culture and UVB irradiation

HaCaT cells (ATCC, Rockville, MD) were cultured in MEM medium supplemented with 10% foetal bovine serum (FBS), penicillin (100 μg/mL), and streptomycin (100 μg/mL) and incubated at 37°C under 5% CO_2_ in a humidified atmosphere.

The cells were irradiated in culture plates placed under a Philips TL 40W/12 RS UVB lamp (Holland) emitting a continuous spectrum between 270 nm and 400 nm, with a peak emission at 313 nm.

### 2.4 RNA interference in vitro

For RNA interference, HaCaT cells were transfected with control scramble siRNA (Santa Cruz Biotechnology), *YAP* siRNA (Santa Cruz Biotechnology), *ERK1* siRNA (Santa Cruz Biotechnology). Transfection was started by using the Lipofectamine RNAiMAX Transfection Reagent (Thermo Fisher Scientific) in Opti‐MEM (Gibco) for 12 h at first, then the medium was changed to complete MEM for another 12 h to get ready for further experiments.

### 2.5 Histology and immunohistochemistry (IHC)

Haematoxylin and eosin (H&E) staining was used to assess the skin epidermal thickness. Mouse skin tissues were fixed in 10% formalin, embedded in paraffin and sectioned to 5 μm slides. The slides were deparaffinized and stained by a Haematoxylin–Eosin Staining Kit (Solarbio) according to the manufacturer's protocol. For IHC staining, skin was subjected to deparaffinization and antigen retrieval at first, then the non‐specific antibody binding was blocked by using 10% bovine serum albumin (BSA; Biosharp) at room temperature for 1 h. After incubating with primary antibodies of FGF10 (ABN44, Millipore, 1:1000 dilution), PCNA (ab29, Abcam, 1:1000 dilution), YAP (ab205270, Abcam, 1:2000 dilution) or Col1a1 (ab138492, Abcam, 1:1500 dilution) at 4°C overnight, appropriate secondary antibodies conjugated with HRP were added and incubated at room temperature. A Metal Enhanced DAB Substrate Kit (Solarbio) was used to visualize the sections followed by haematoxylin counterstaining. All images were captured by using a Nikon ECLIPSE Ni microscope.

### 2.6 Immunofluorescence

For in vitro experiments, HaCaT cells were first fixed in 4% paraformaldehyde for 15 min and then permeabilized in 0.5% Triton X‐100 for 15 min at room temperature. Cells were then blocked by 5% BSA, incubated with the primary antibodies at 4°C overnight and then incubated with fluorescent‐labelled secondary antibodies for 1 h. Finally, the cell nuclei were stained with DAPI. Images were visualized and captured by using a Leica SP8 confocal microscope.

### 2.7 Western blot

RIPA lysis buffer (Thermo Fisher Scientific) with Protease and Phosphatase Inhibitor Cocktail (Abcam) was used to extract total proteins from fresh skin tissues and cell samples. Protein concentrations were detected by using the Pierce BCA Protein Assay Kit (Thermo Fisher Scientific), and proteins were separated by SDS‐PAGE and transferred to PVDF membrane (Millipore) followed by blocking with 5% non‐fat milk (BD Bio‐sciences). After incubation with primary and secondary antibodies, each blot was developed by using the ECL regent (Millipore) and captured by Amersham Image 600 system (GE Healthcare Life Sciences). Antibodies used in the western blot are listed in Table S1.

### 2.8 Quantitative RT‐PCR

TRIzol Reagent (Invitrogen) was used to extract total RNA from cells or fresh skin tissues. Complementary DNA was reverse transcribed by using the GoScript Reverse Transcription System (Promega), and quantitative RT‐PCR was performed with SYBR Green PCR Master Mix (Thermo Fisher Scientific) following the manufactures' protocols. The mRNA levels were normalized to β‐actin mRNA level. The primer sequences of the target genes are shown in Table S2.

### 2.9 Cell viability study

A cell count kit‐8 (CCK‐8, Beyotime, China) was employed in this experiment to quantitatively evaluate HaCaT viability. Briefly, approximately 1 × 10^4^ cells were seeded on each film placed in the 24‐well plates for 24 h. Approximately 900 μl serum‐free DMEM medium and 100 μl CCK‐8 solution were added to each sample, followed by incubation at 37°C for several time. Supernatant was transferred to 96‐well plate, the optical density (OD) at 450 nm was determined using a microplate reader (Multiskan MK33, Thermo lab systems, Finland).

### 2.10 Statistical analysis

Data analysis was done by GraphPad Prism 8 Software, and Student's *t*‐test was used to determine significance. A *P*‐value <0.05 was considered significant. All data shown in this study are a mean ± SEM and a repeat of at least three times unless and otherwise indicated.

## 3 RESULTS

### 3.1 FGF10 is upregulated following UVB‐induced skin injury

**FIGURE 1:**
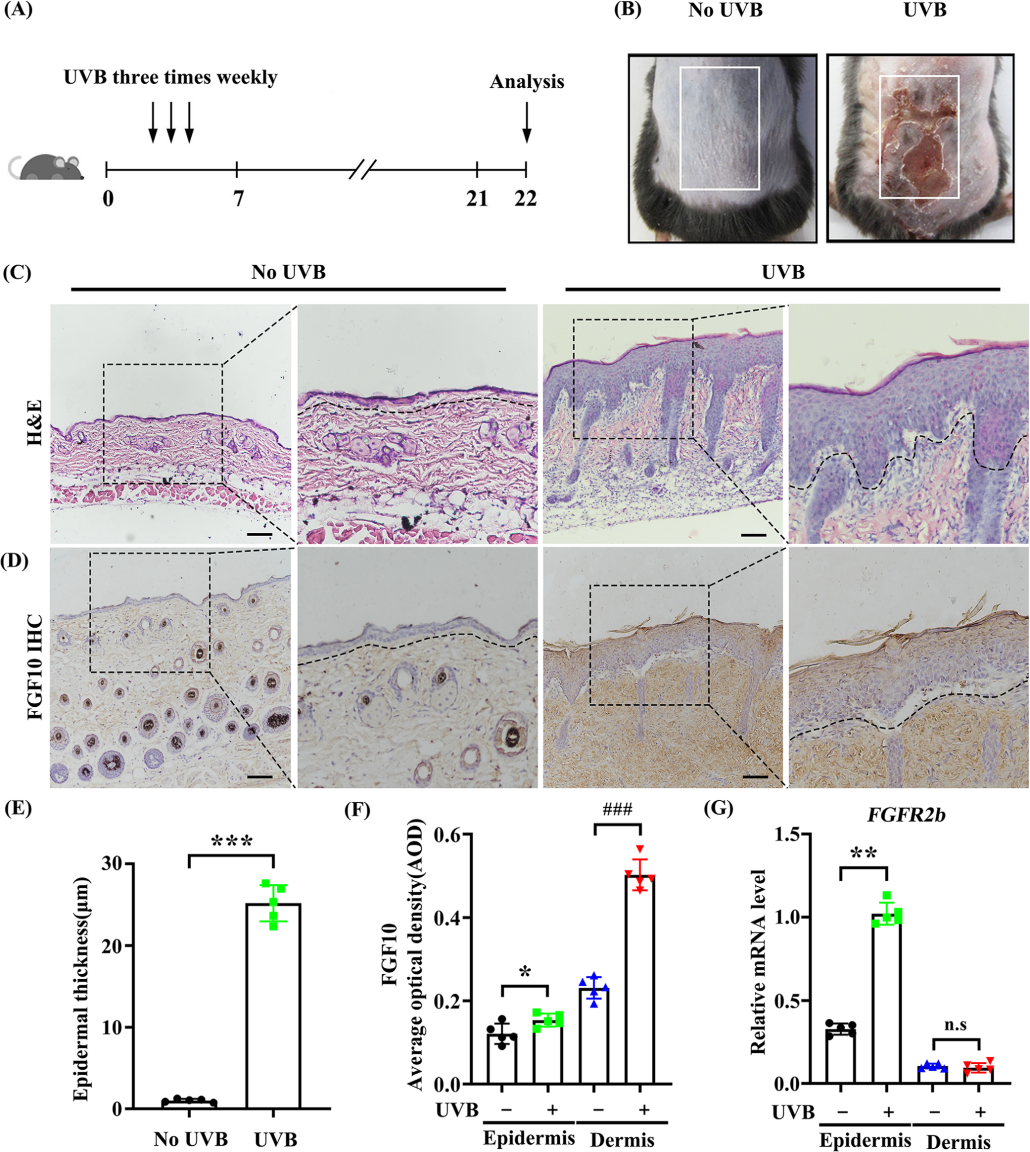
FGF10 is upregulated in UVB‐induced skin injury. (A) The method of UVB radiation treatment in C57BL/6J mice experimental model. (B) Skin injury induced by UVB radiation. Skin of wild‐type (WT) C57BL/6J mice (Left). Skin of UVB radiation C57BL/6J mice (Right). (C) HE staining of wild‐type (WT) C57BL/6J mice and UVB radiation C57BL/6J mice. (*n* = 5). Scale bars = 100 μm. (D) Immunohistochemical staining of wild‐type (WT) C57BL/6J mice and UVB radiation C57BL/6J mice. The positive staining (brown) demonstrated positive expression (*n* = 5). Scale bars = 100 μm. (E) Quantification of epidermal thickness from (C). (F) Quantification of AOD from (D). (G) Quantified by quantitative real‐time PCR (qRT‐PCR) in untreated skin and UVB treated skin. The skin of epidermis and dermis were counted separately (*n* = 5). The data are presented as the means ± SEM. ^###^ *P* < 0.001, ***P < 0.001, **P < 0.01, *P < 0.05, vs. the corresponding UVB untreated group; N.S., nonsignificant

To analyse the relationship between FGF10 and UVB‐induced skin injury, we assessed the protein expression of FGF10 in an animal model for UVB damage. Male mice (8–10 weeks) were exposed to UVB at an intensity of 200 mJ/cm^2^ for 3 weeks (Figure 1A). We found that the dorsal skin of irradiated mice became rough, wrinkled, red, swollen, and crusted, indicating the induction of skin damage (Figure 1B). HE staining revealed that UVB radiation increased epidermal thickness (Figure 1C,E). Meanwhile, IHC staining showed that collagen type I alpha 1 (Col1a1) production was prevented (Figure S1A). As expected, the expression of FGF10 was significantly increased by UVB irradiation, compared with un‐irradiated mice (Figure 1D,F). As a paracrine factor, FGF10 can be secreted by multiple organs, including skin, lung, and kidney.^22^ Interestingly, we found that FGF10 is highly expressed in the dermis.

In addition, FGF10/FGFR2b‐mediated pathway has been reported to be involved in signal transduction between mesenchymal cells and epithelial cells, which plays an important role in various tissues.^23^ Thereby, to verify this possibility, we separated the UVB‐irradiated mouse skin to epidermis and dermis. We found that *FGFR2b* mRNA level was upregulated in the epidermis following UVB treatment, but not in the dermis (Figure 1G). Together, these data indicate that FGF10 is upregulated in UVB‐induced skin dermal fibroblasts and acts mainly on keratinocytes.

### 3.2 FGF10 overexpression alleviates UVB‐induced skin injury

**FIGURE 2:**
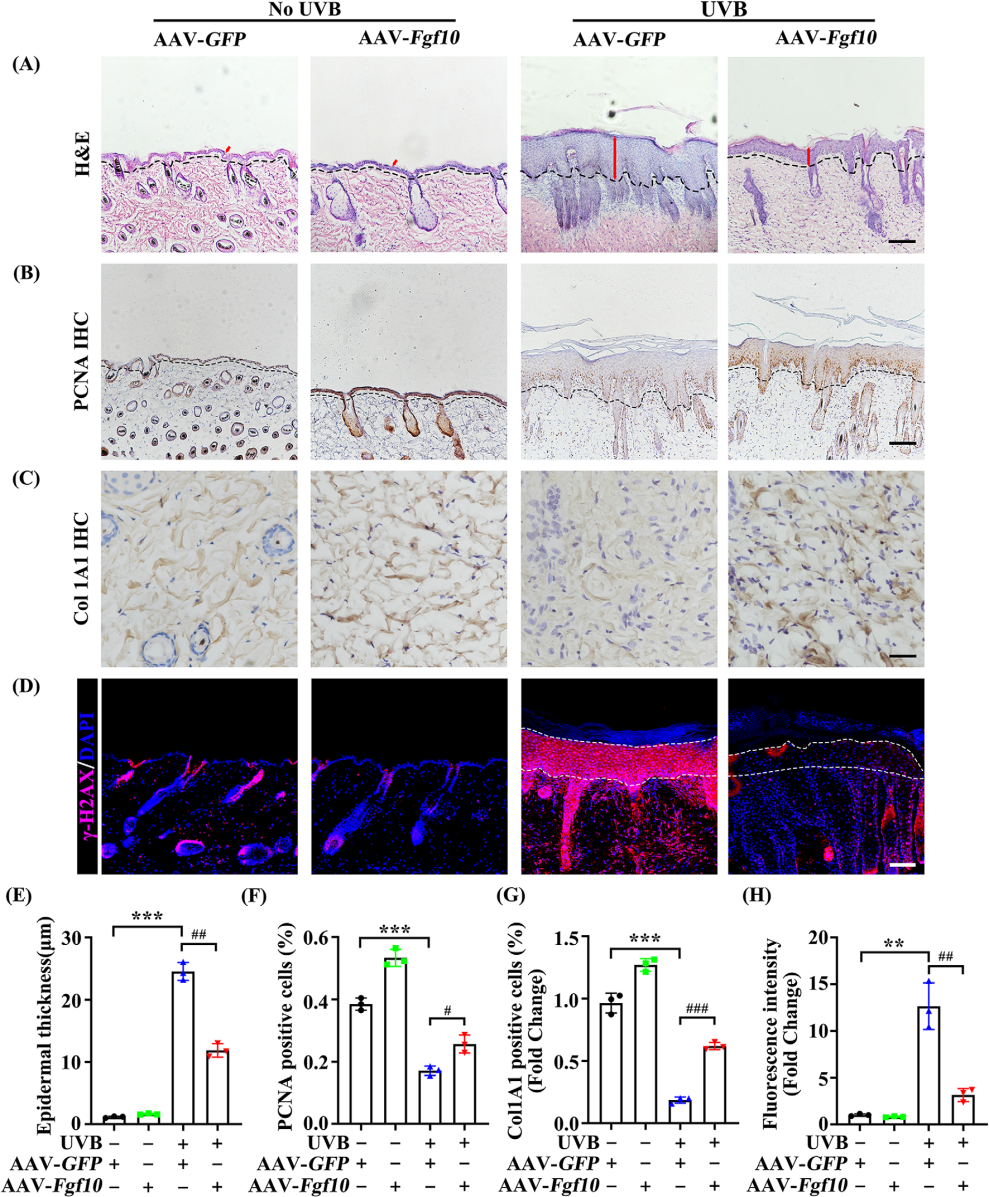
FGF10 overexpression alleviates UVB‐induced skin injury. (A) HE staining of AAV‐*GFP* or AAV‐*Fgf10* treated with or without UVB (*n* = 3). Scale bars = 100 μm. (B) Immunohistochemical staining of PCNA in the skin of AAV‐*GFP* or AAV‐*Fgf10* treated with or without UVB. The positive staining (brown) demonstrated positive expression (*n* = 3). Scale bars = 100 μm. (C) Representative IHC of Col1a1 in the skin of AAV‐*GFP* or AAV‐*Fgf10* treated with or without UVB. The positive staining (brown) demonstrated positive expression (*n* = 3). Scale bars = 20 μm. (D) Immunofluorescence staining of γ‐H2AX in the skin of AAV‐*GFP* or AAV‐*Fgf10* treated with or without UVB (*n* = 3). Scale bars = 100 μm. (E) Quantification of epidermal thickness from (A). (F) Quantification of AOD from (B). (G) Quantification of AOD from (C). (H) Quantification percentage of γ‐H2AX‐positive from (D). The data were presented as the means ± SEM. ***P < 0.001, **P < 0.01, ^###^ *P* < 0.001, ^##^ *P* < 0.01, ^#^ *P* < 0.05, vs. the corresponding UVB untreated AAV‐*GFP* group

To investigate the role of FGF10 in UVB‐induced epidermal injury, we utilized an AAV‐mediated gain construct (Figure S2A). It should be noticed that the AAV transfection induced FGF10 overexpression was markedly observed in skin (Figures S2B, S2C). UVB‐induced increases in dorsal skin epidermal thickness were reduced by FGF10 overexpression (Figure 2A,E), and the density of proliferating cell nuclear antigen (PCNA) was significantly increased under the same conditions (Figure 2B,F). It has been reported that severe UVB damage reduces collagen secretion.^24^, ^25^ Consistently, the expression of Col1a1, which is required for skin elasticity, was increased by FGF10 overexpression (Figure 2C,G), indicating that FGF10 can restore collagen secretion following UVB damage. Immunofluorescence staining showed that DNA damage response protein γ‐H2AX accumulation following UVB irradiation was reduced by FGF10 overexpression, suggesting a reduction in the level of DNA damage (Figure 2D,H). Together, these data suggest that FGF10 plays a protective role during UVB‐induced skin injury.

### 3.3 YAP is downregulated during UVB‐induced skin injury

**FIGURE 3:**
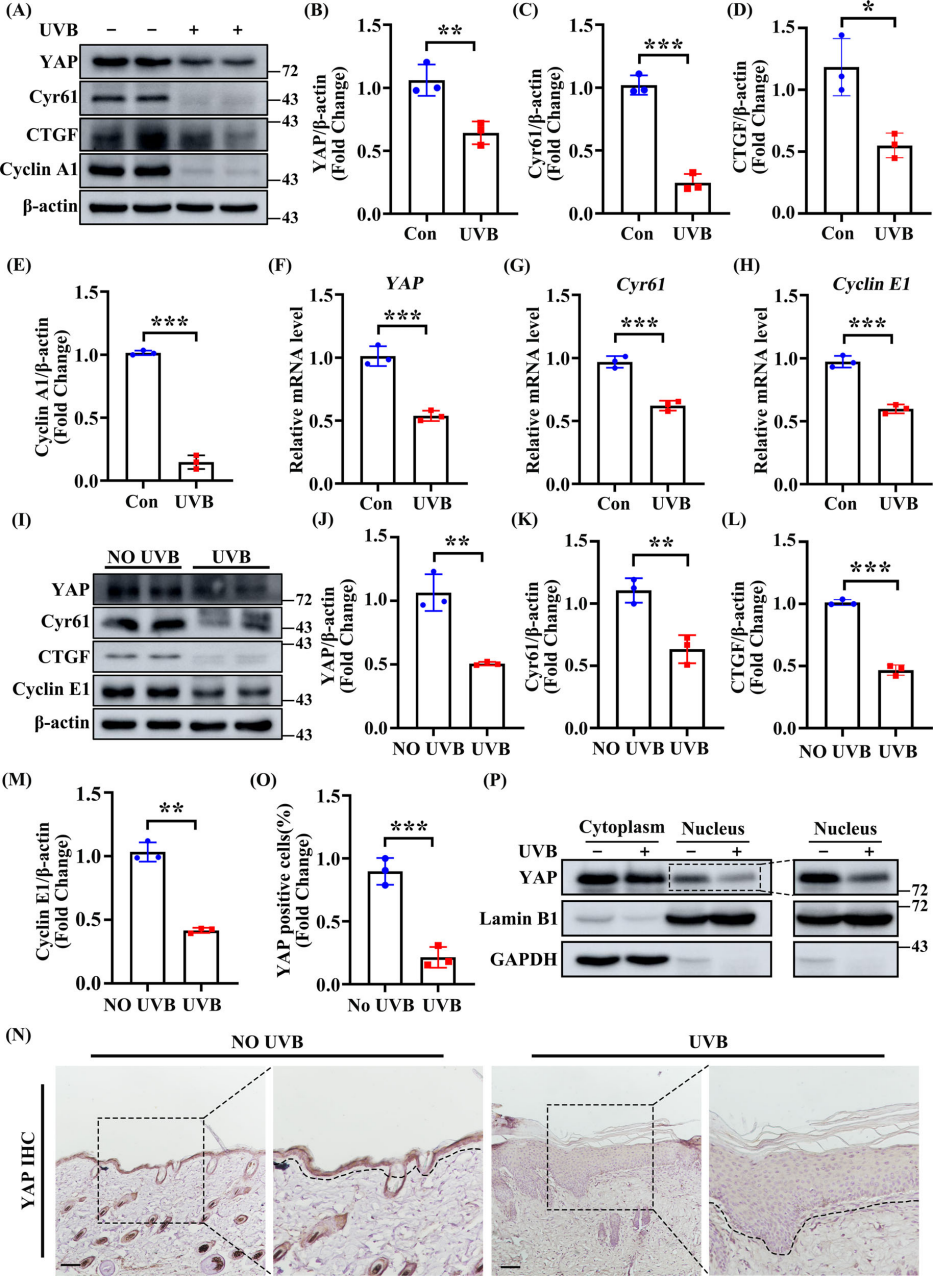
YAP is downregulated during UVB‐induced skin injury. (A) The expression levels of YAP, Cyr61, CTGF, Cyclin A1 in the HaCaT subjected to untreated or UVB‐irradiated (*n* = 3). β‐Actin was used as a loading control. (B–E) Quantification of YAP, Cyr61, CTGF, Cyclin A1 levels in (A). (F–H) The mRNA levels of *YAP*, *Cyr61*, *Cyclin E1* were quantified by quantitative real‐time PCR (qRT‐PCR) in untreated or UVB treated HaCaT (*n* = 3). (I) Protein expression levels of YAP, Cyr61, CTGF, Cyclin E1 in the skin tissues subjected to untreated or UVB‐irradiated. (J–M) Quantification of YAP, Cyr61, CTGF, Cyclin A1 levels in (I). (N) Representative IHC staining of YAP in the epidermal of skin after untreated or UVB‐irradiated (*n* = 3). Scale bars = 100 μm. (O) Quantification the percentage of YAP‐positive cells from (N). (P) Western blot analysis the subcellular localization of YAP in untreated or UVB treated HaCaT (*n* = 3). The data are presented as the means ± SEM. ***P < 0.001, **P < 0.01, vs. the corresponding untreated group

It has previously been reported that ultraviolet radiation can inhibit cell proliferation and induce cell cycle arrest.^26^, ^27^ Western blot analysis showed that the expression of Cyclin A1 and YAP, a protein involved in both proliferation and cell cycle, together with its downstream transcription target proteins Cyr61 and CTGF were markedly decreased in HaCaT cells following UVB irradiation (Figure 3A–E). Furthermore, UVB irradiation significantly decreased the transcription levels of *YAP* and its downstream transcription target gene *Cyr61*, as well as proliferation marker *Cyclin E1* (Figure 3F–H). Similar data were obtained from in vivo experiments (Figure 3I–O). Acting as a nuclear transcription factor, we observed that the nuclear accumulation of YAP was greatly reduced by UVB (Figure 3P). These in vitro and in vivo data show that UVB damage influences the expression of YAP in the skin epidermis.

### 3.4 FGF10 protects against UVB‐induced cell damage via a YAP‐dependent pathway

**FIGURE 4:**
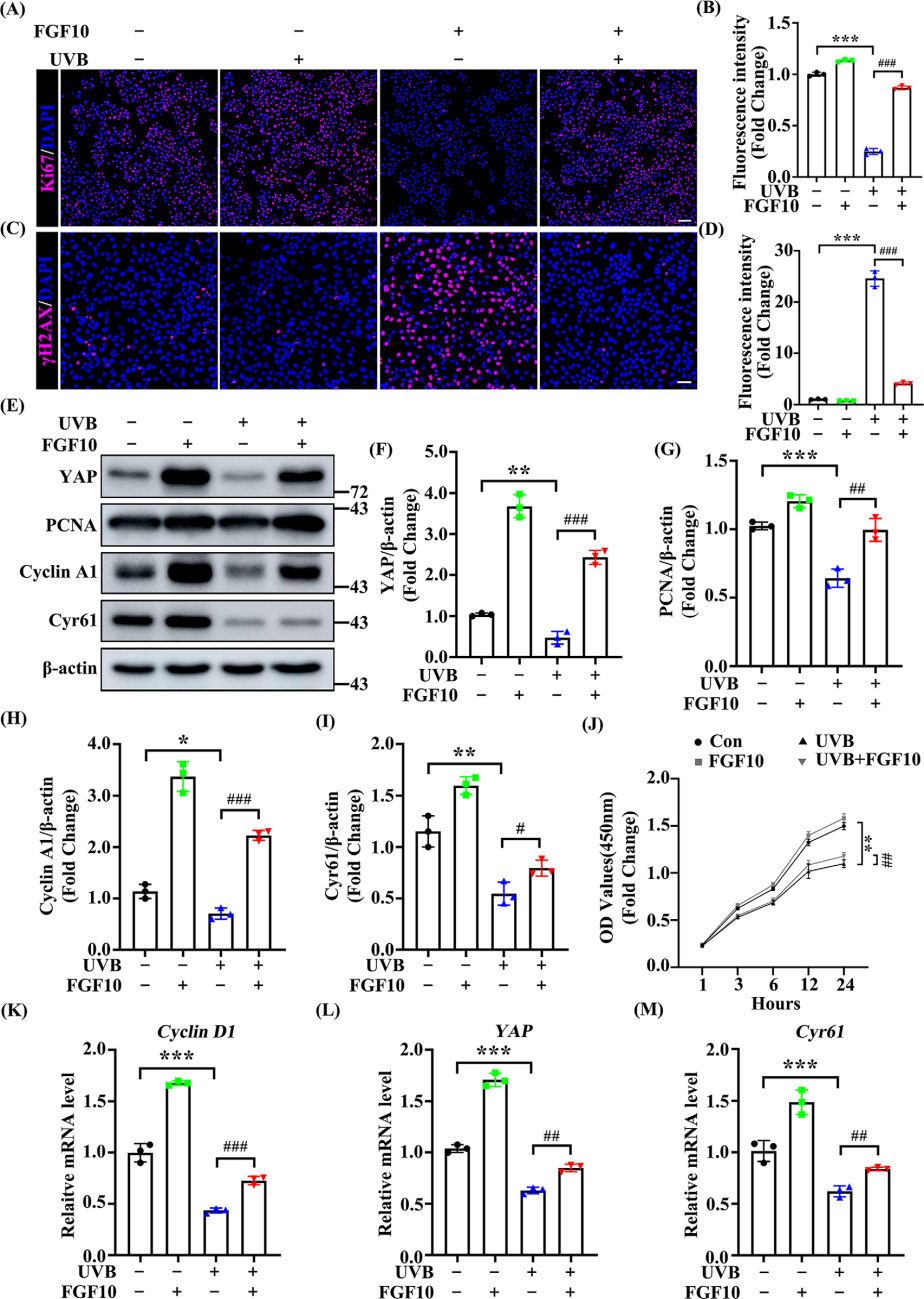
FGF10 protects against UVB‐induced cell damage via a YAP‐dependent pathway. (A) Immunofluorescence staining of Ki‐67 (Red) in untreated or UVB treated HaCaT with or without FGF10. Nuclei were stained with DAPI (Blue) (*n* = 3). Scale bars = 100 μm. (B) Quantification the percentage of Ki‐67‐positive cells from (A). (C) Immunofluorescence staining of γ‐H2AX (Red) in untreated or UVB treated HaCaT with or without FGF10. Nuclei were stained with DAPI (Blue) (*n* = 3). Scale bars = 50 μm. (D) Quantification the percentage of γ‐H2AX‐positive cells from (C). (E) The protein expression of YAP, PCNA, Cyclin A1 and Cyr61 were measured by immunoblotting assay in untreated and UVB treated with or without FGF10. β‐actin was used as a loading control (*n* = 3). (F–I) Quantification of YAP, PCNA, Cyclin A1 and Cyr61 protein levels in (E). (J) CCK8 assays were used to estimate the cell proliferation at different time points. (K–M) The mRNA levels of *YAP*, *PCNA*, *Cyclin A1* and *Cyr61* were quantified by quantitative real‐time PCR (qRT‐PCR) in untreated and UVB treated HaCaT (*n* = 3). The data are presented as the means ± SEM. ***P < 0.001, **P < 0.01, *P < 0.05, ^###^ *P* < 0.001, ^##^ *P* < 0.01 vs. the corresponding untreated group

FGF10 is involved in proliferation, cell development, and the restoration of cell growth.^28^ To further address the role for FGF10 in the response to UVB‐induced epidermal injury, we examined the proliferation marker Ki‐67 and DNA damage repair indicator γ‐H2AX by immunofluorescence. We revelled that FGF10 treatment largely counteracted UVB‐irradiation induced proliferation inhibition and DNA damage as reflected by Ki‐67 and γ‐H2AX staining (Figure 4A–D). Furthermore, FGF10 significantly increased the expression of PCNA and Cyclin A1, another two proliferation markers, in UVB‐irradiated cells (Figure 4E,G,H). We next used the CCK‐8 assay to examine cell number following UVB damage, and found that FGF10 overexpression rescued cell survival compared with control (Figure 4J). In addition, we examined the expression of cell cycle markers by qPCR, finding that *Cyclin D1* expression was markedly increased by FGF10 overexpression (Figure 4K). Together, these data suggest that FGF10 facilitates cell survival and proliferation in response to UVB‐induced damage by an unknown mechanism.

Since YAP controls the proliferation of cells, tissues and organs by regulating the cell cycle,^29^, ^30^ and YAP expression is decreased following UVB irradiation, we therefore hypothesized that there might be a connection between FGF10 and YAP. As shown in Figure 4E, the expression of YAP and its target protein Cyr61 were increased by FGF10 treatment, suggesting that FGF10 induces YAP activity following UVB irradiation (Figure 4F,I). Meanwhile, *YAP* and *Cyr61* transcription were upregulated by FGF10 overexpression (Figure 4L,M). Taken together, these results indicate that FGF10 improves epidermal proliferation following UVB‐induced skin injury, possibly via YAP.

### 3.5 The FGF10‐dependent response to UVB‐induced skin injury requires YAP activity

**FIGURE 5:**
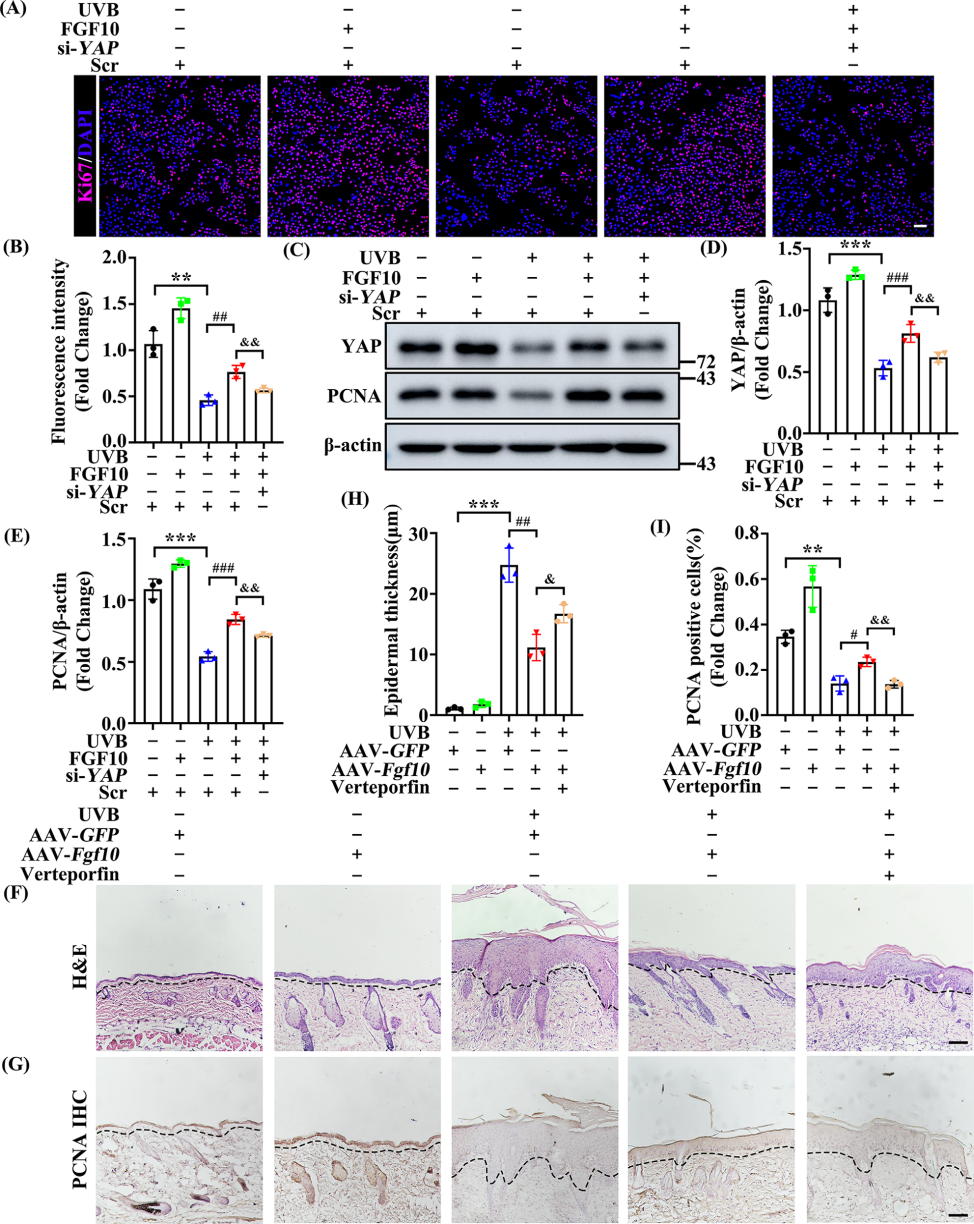
The FGF10‐dependent response to UVB‐induced skin injury requires YAP activity. (A) Immunofluorescence staining of Ki‐67 (Red) in untreated or UVB treated HaCat with or without FGF10 treatment in the presence or absence of si‐*YAP*. Nuclei were stained with DAPI (blue) (*n* = 3). Scale bars = 100 μm. (B) Quantification the percentage of Ki‐67‐positive cells from (A). (C) The expression of YAP and PCNA was measured by immunoblotting assay in untreated or UVB treated HaCaT with FGF10 or without FGF10 treatment in the presence or absence of si‐*YAP*. β‐Actin was used as a loading control (*n* = 3). (D,E) Quantification of YAP, PCNA protein levels in (C). (F) Representative HE staining of AAV‐*GFP* or AAV‐*Fgf10* transfected skin treated with or without UVB in the presence or absence of VP (*n* = 3). Scale bars = 100 μm. (H) Quantification the epidermal thickness from (F). (G) Immunohistochemical staining of PCNA in the AAV‐*GFP* or AAV‐*Fgf10* transfected skin treated with or without UVB in the presence or absence of VP. The positive staining (brown) demonstrated positive expression (*n* = 3). Scale bars = 100 μm. (I) Quantification the epidermal thickness from (G). The data are presented as the means ± SEM. ***P < 0.001, **P < 0.01, *P < 0.05, ^###^ *P* < 0.001, ^##^ *P* < 0.01, ^&&^ *P* < 0.01, ^&^ *P* < 0.05 vs. the corresponding control siRNA HaCaT group

We next sought to determine how FGF10 regulates the proliferation of keratinocytes following UVB treatment. Considering that UVB treatment downregulates the activity of YAP in the epidermis, we speculated that FGF10 may alleviate UVB‐induced injury by upregulating YAP activity. To test our hypothesis, we first treated HaCaT with *YAP* small‐interfering RNA (si‐*YAP*) to silence YAP expression and examined proliferation by Ki‐67 staining (Figure S3A). We found that the protective effect of FGF10 on proliferation following UVB treatment was abolished by *YAP* siRNA (Figure 5A,B). Meanwhile, the protein expression of YAP and PCNA both markedly decreased following *YAP* siRNA transfection (Figure 5C–E). To further validate the positive functions of YAP on epidermal keratinocytes, we used the YAP/TEAD interaction inhibitor Verteporfin (VP). VP treatment induced a marked thickening of the epidermis in lesion areas (Figure 5F,H), and an obvious downregulation of proliferation in the epidermis, as determined by PCNA IHC staining (Figure 5G,I). Taken together, these data demonstrate that the FGF10‐dependent protection against UVB‐induced injury is YAP‐dependent.

### 3.6 FGF10 protects UVB‐induced skin injury through the ERK‐YAP pathway

**FIGURE 6:**
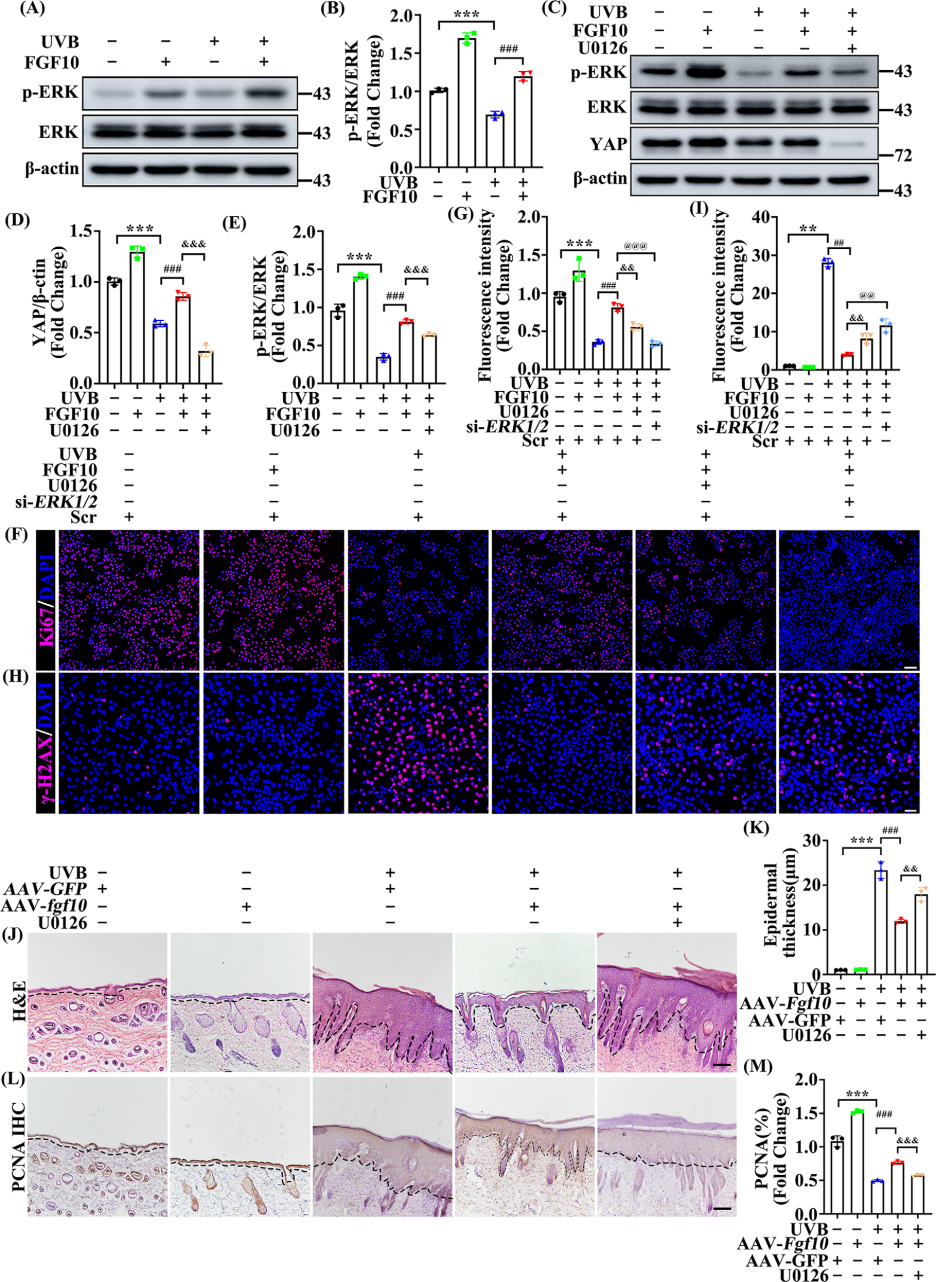
FGF10 protects UVB‐induced skin injury through the ERK‐YAP pathway. (A) The expression levels of p‐ERK and ERK in the untreated or UVB treated with or without FGF10. β‐Actin was used as a loading control (*n* = 3). (B) Quantification of p‐ERK/ERK levels in (A). (C) The protein expression of p‐ERK, ERK and YAP were measured by immunoblotting assay in untreated or UVB treated with or without FGF10 in the presence or absence of U0126. β‐Actin was used as a loading control (*n* = 3). (D,E) Quantification of YAP, p‐ERK/ERK levels in (C). (F) Immunofluorescence staining of Ki‐67 (Red) in the untreated or UVB treated HaCaT with or without FGF10 in the presence or absence of U0126 and si‐*ERK1/2*. Nuclei were stained with DAPI (Blue) (*n* = 3). Scale bars = 100 μm. (G) Quantification the percentage of Ki67‐positive cells from (F). (H) Immunofluorescence staining of γ‐H2AX (Red) in the untreated or UVB treated HaCaT with or without FGF10 in the presence or absence of U0126 and si‐*ERK1/2*. Nuclei were stained with DAPI (Blue) (*n* = 3). Scale bars = 50 μm. (I) Quantification the percentage of γ‐H2AX‐positive cells from (H). (J) Representative HE staining of AAV‐*GFP* or AAV‐*Fgf10* transfected skin treated with or without UVB in the presence or absence of U0126 (*n* = 3). Scale bars = 100 μm. (K) Quantification the epidermal thickness from (J). (L) Immunohistochemical staining of PCNA in the AAV‐*GFP* or without UVB and AAV‐*Fgf10* transfected skin treated with or without UVB in the presence or absence of U0126. The positive staining (brown) demonstrated positive expression (*n* = 3). Scale bars = 100 μm. (M) Quantification the PNCA‐positive from (L). The data are presented as the means ± SEM. ***P < 0.001, **P < 0.01, ^###^ *P* < 0.001, ^##^ *P* < 0.01, ^&&&^ *P* < 0.001, ^&&^ *P* < 0.01, ^@@@^ *P* < 0.001, ^@@^ *P* < 0.01 vs. the corresponding untreated group

ERK is an extracellular regulatory protein kinase that functions downstream of the FGF10 receptor FGFR2.^30^, ^31^ In addition, the ERK‐YAP pathway is reported to regulate the cell cycle, cell proliferation, and tissue repair.^32^, ^33^ Thus, we speculated that FGF10 may mitigate UVB damage through ERK‐YAP signalling activity. FGF10 treatment significantly increased the level of ERK phosphorylation (Figure 6A,B), indicating that FGF10 upregulates the activity of ERK following UVB‐induced injury. To further examine whether FGF10 regulates YAP activity via ERK, we treated cells with the ERK specific inhibitor U0126 and examined YAP expression. Following this treatment, we found that UVB‐dependent phosphorylation of ERK and YAP expression were both decreased (Figure 6C–E). To further investigate the role of ERK in FGF10 protects keratinocytes proliferation, we utilized ERK‐specific interfering RNA to verify (Figure S4A). Moreover, Ki‐67 staining showed that U0126 or si‐*ERK1/2* treatment restrained proliferation compared with the control group (Figure 6F,G). γ‐H2AX staining observed that silence of *ERK1*/*2* counteracted the protection of FGF10 on DNA damage (Figure 6H,I). These in vitro assays suggest that ERK activity is essential for FGF10 regulation of YAP expression following UVB irradiation. To confirm this result in vivo, we examined epidermal skin treated with U0126. HE staining showed that U0126 further increased epidermal thickening induced by UVB treatment (Figure 6J,K). Similarly, IHC staining indicated that U0126 blocked the protective effect of FGF10, resulting in weakened proliferation as indicated by decreased expression of PCNA (Figure 6L,M). Together, our results show the functional importance of ERK in FGF10‐mediated activation of YAP following UVB‐induced skin injury.

## 4 DISCUSSION

Ultraviolet radiation can cause pathological reactions in the epidermis, such as the inhibition of proliferation and thickening of the epidermis,^34^ which are detrimental to the daily life of affected individuals. However, the molecular mechanisms underpinning the induction and repair of such damage is poorly understood.

Cells and tissues have evolved many methods to alleviate the injury caused by UVB. FGFs play a key role in these methods, promoting keratinocytes proliferation and repair.^35^ FGF10 is a mesenchymal signalling protein found in epithelial cells, which plays a vital role in organ development. In this study, we have shown that UVB damage induces a stress response that elevates the secretion of FGF10 in the dermis, while the corresponding receptors at the epidermal damage site cooperate to alleviate injury. It is further demonstrated that overexpression of FGF10 in mice significantly protects the epidermis from UVB damage, as evidenced by diminished epidermal thickness, increased keratinocyte proliferation, and decreased DNA damage. For the first time, we show that FGF10 is an important mediator of a tissue and cellular response to UVB.

As a transcriptional co‐activator of the Hippo pathway, YAP participates in the proliferation and differentiation of cells, and regulates the growth and development of the body.^36^ Furthermore, it has been shown that YAP promotes proliferation of keratinocytes at skin wounds, thereby promoting wound healing.^37^ We have revealed that keratinocytes proliferation and YAP activity were significantly inhibited by UVB treatment, these were largely restored by FGF10 administration. Loss of ERK/YAP activity by siRNA or small molecule inhibitors disrupted FGF10‐dependent protection, indicating that the protective effect of FGF10 on UVB‐mediated skin injury is ERK/YAP‐dependent.

Acting as a direct downstream target of FGF10, ERK binds to FGFR2 and mediates a signal cascade to induce a series of cellular functions.^38^ Accordingly, we found that ERK inhibitor U0126 and ERK siRNA blocked FGF10‐induced YAP activation, aggravated epidermal thickness, and decreased proliferation of the epidermis accumulation following UVB treatment. Together, our results showed that ERK plays indispensable roles in the FGF10‐mediated response to UVB‐induced skin injury.

In summary, our study demonstrates the protective effects of FGF10 in UVB‐mediated epidermal injury. FGF10 and the subsequent ERK‐dependent YAP activation contribute to epidermal repair and may be essential for the maintenance of skin homeostasis of regulation of UVB‐induced skin injury. These findings could broaden our understanding of the regulatory role of FGF10 in the epidermis, indicating a promising new avenue for ameliorating UVB radiation damage.

## 5 CONCLUSION

Our data indicate that FGF10 can alleviate UVB‐induced skin damage by promoting keratinocytes proliferation through ERK/YAP, providing a mechanism study for the treatment of UVB damage.

## AUTHOR CONTRIBUTIONS

Nan Wang, Yetong Dong, Xiejun Xu, Litai Jin and Weitao Cong conceived, designed and supervised the study. Nan Wang, Yingjie Shen, Zhiyuan Huang and Yin Yu researched the data. Nan Wang, Zhili Liu, Wenjie Gong, Siyi Zhang, Yeyi Zheng, Yonghuan Song and Zhongxin Zhu contributed to the discussion and design of the project. Nan Wang, Litai Jin and Weitao Cong wrote the paper. Nan Wang and Yetong Dong are the guarantor of this work and, as such, had full access to all the data in the study and takes responsibility for the integrity of the data and the accuracy of the data analysis. All authors read and approved the final manuscript.

## CONFLICT OF INTEREST

The authors declare no competing interests.

## Supporting information


**Appendix S1** Supporting Information

## Data Availability

Data supporting the findings of this study are available from the corresponding author upon reasonable request.
