# Supplementary material for: Fibroblast growth factor 10 protects against UVB‐induced skin injury by activating the ERK/YAP signalling pathway
Source: Cell Prolif. 2022 Jul 18;55(11):e13315. doi: 10.1111/cpr.13315 (PMC9628220; doi:10.1111/cpr.13315)
Supplement: Supplementary file 1 — Appendix S1 Supporting Information [file CPR-55-e13315-s001.docx]

**SUPPLEMENTARY MATERIAL**

**Fibroblast growth factor 10 protects against UVB-Induced Skin Injury by Activating the ERK/YAP Signaling Pathway**

**AUTHORS**

Nan Wang, **Yetong Dong, Xiejun Xu, Yingjie Shen, Zhiyuan Huang, Yin Yu, Zhili Liu, Wenjie Gong, Siyi Zhang, Yeyi Zheng, Yonghuan Song, Zhongxin Zhu, Litai Jin, Weitao Cong.**

**This file includes the following subsections:**

**Supplementary materials;**

**Supplementary Figures S1-S4;**

**Supplementary Table S1;**

**Supplementary Table S2;**

**SUPPLEMENTARY MATERIALS**

**SUPPLEMENTARY FIGURES**


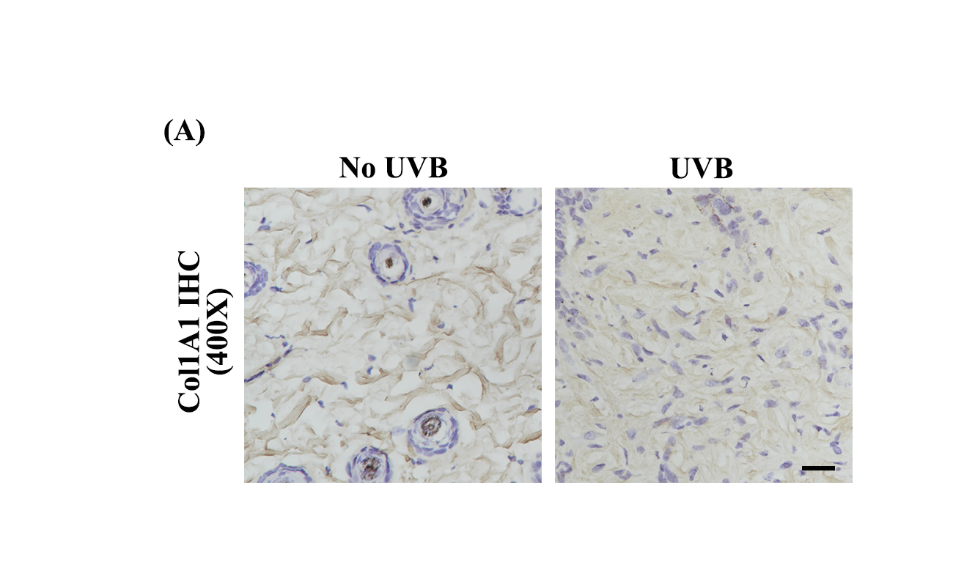


**Supplementary Figure S1: Identification of the skin injury by UVB-radiation.** (A) Immunohistochemical staining of Col1a1 in the skin of treated with or without UVB (n = 3). Scale bars = 50 μm.


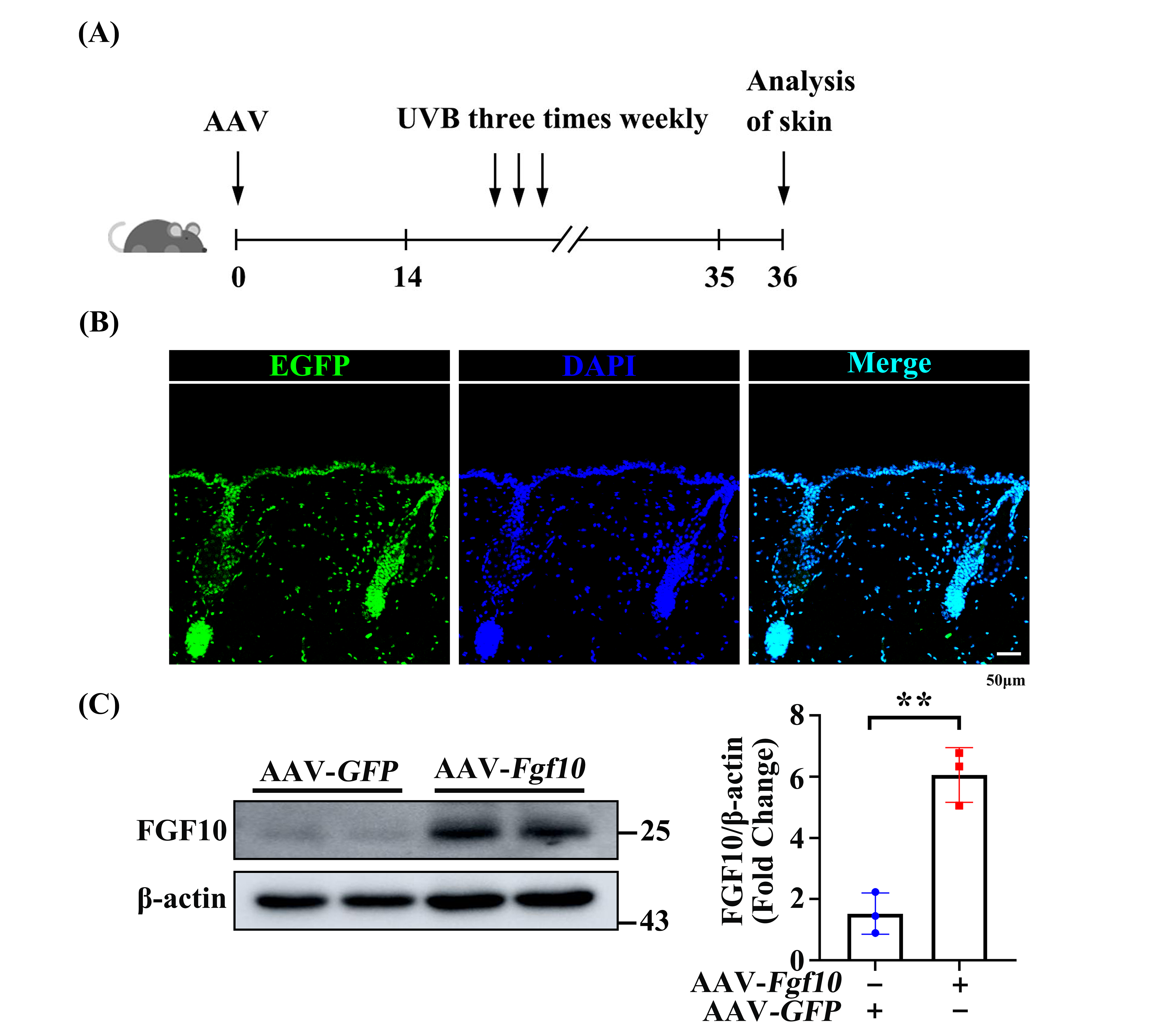


**Supplementary Figure S2: Identification of AAV-mediated gene delivery in mice. (**A**)** The time schedule of mice subjected to AAV transfection then treated UVB-radiation in C57BL/6J mice experimental model. **(**B**)** Representative immunofluorescence staining of EGFP in the skin of mice subjected to AAV transfection. Scale bars = 50 μm. (C) Protein expression levels of FGF10 in the skin of mice subjected to AAV transfection. β-actin was used as a loading control (n = 3).

**
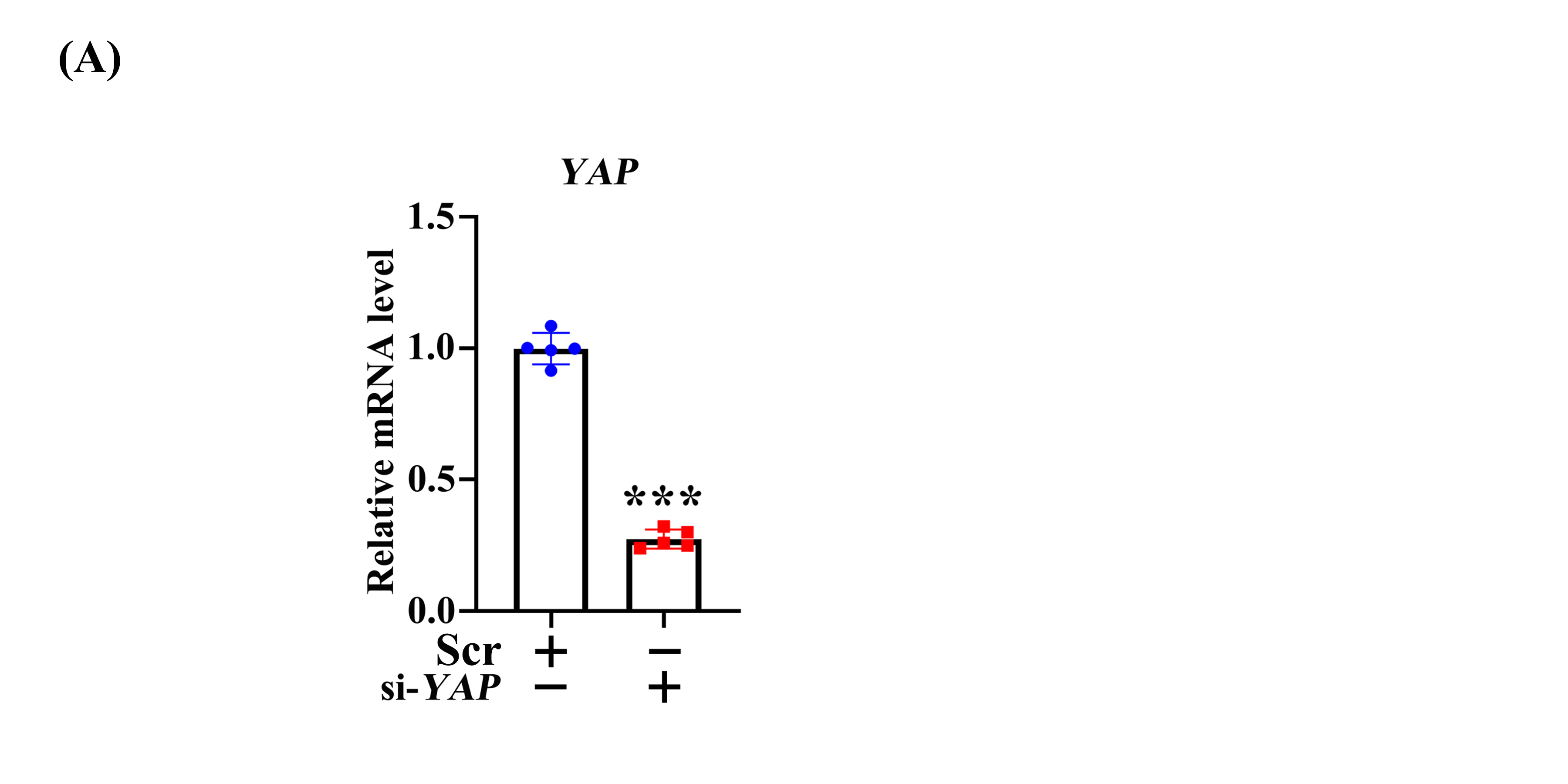
**

**Supplementary Figure S3: Identification the effect of *YAP* gene silencing.** (A) The mRNA level of YAP was quantified by quantitative real-time PCR (qRT-PCR) in untreated or si-*YAP* treated HaCaT (n = 5).

**
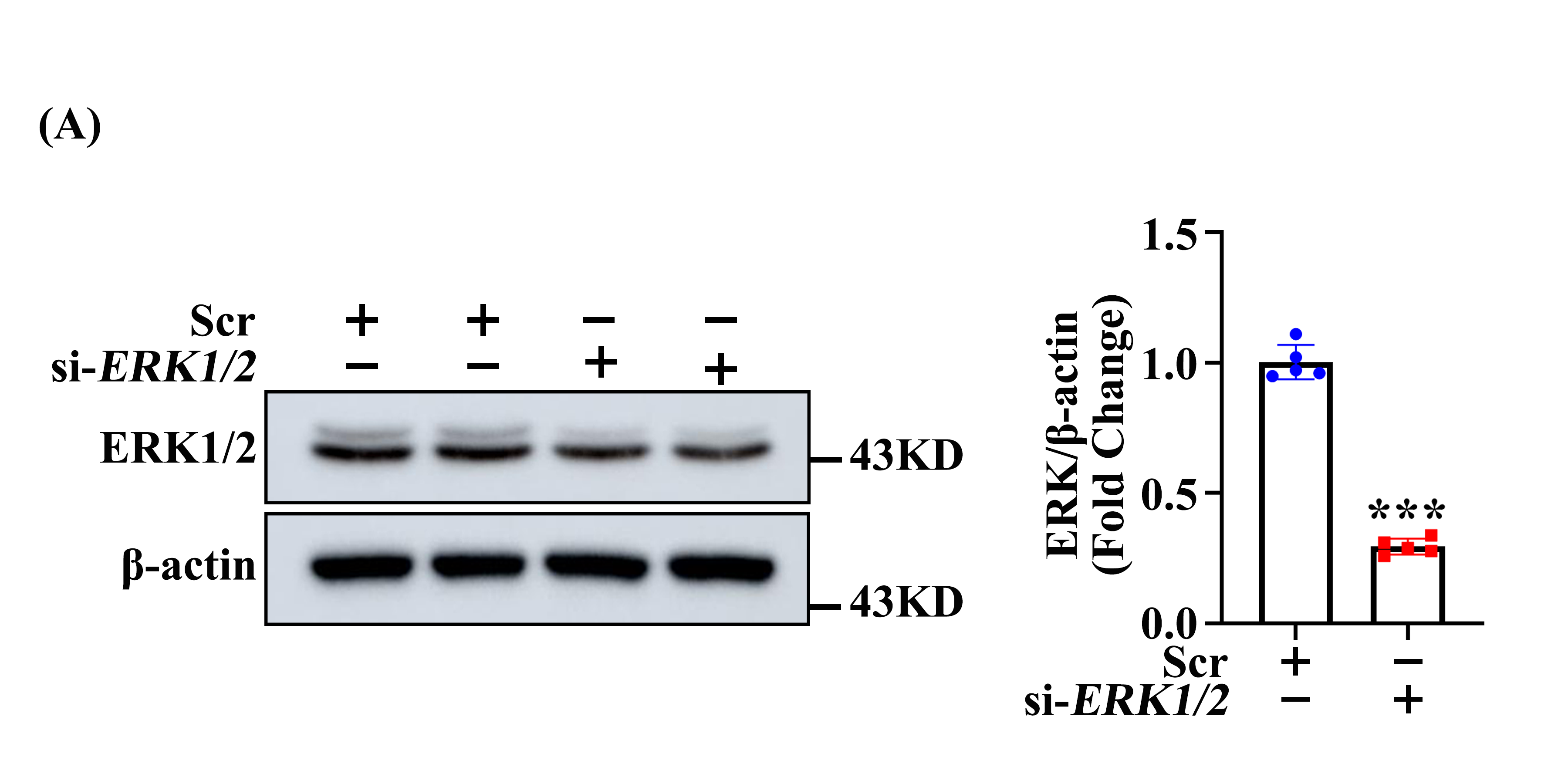
**

**Supplementary Figure S4: Identification the effect of *ERK1/2* gene silencing.** (A) Protein expression level of ERK1/2 in the HaCaT subjected to untreated or si-*ERK1/2*. β-actin was used as a loading control (n = 5).

**Supplementary Table 1. List of antibodies used in the western blot.**

| **Antibody** | **Manufacture** | **Cat No.** | **Dilution** |
| --- | --- | --- | --- |
| FGF10  YAP  Cyr61  p-ERK1/2  ERK1/2  CTGF  PCNA  Cyclin A1  Cyclin D1  Cyclin E1  Ki67  γ-H2AX  β-actin | Millipore  Cell Signaling Technology  Cell Signaling Technology  Cell Signaling Technology  Cell Signaling Technology  Cell Signaling Technology  Abcam  Abclonal  Cell Signaling Technology  Cell Signaling Technology  Cell Signaling Technology  Abclonal  Cell Signaling Technology | ABN44  14074  14479  4370  9102  86641  ab2426  A14527  55506  20808  12075  AP0687  4970 | 1:2000  1:1000  1:1000  1:1000  1:1000  1:1000  1:1000  1:1000  1:1000  1:1000  1:200  1:200  1:1000 |

**Supplementary Table-S2. Primer sequences used for reverse transcription-quantitative PCR.**

| **Gene Name** | **Forward Primer** | **Reverse Primer** |
| --- | --- | --- |
| ***FGF10*** | CAGTAGAAATCGGAGTTGTTGCC | TGAGCCATAGAGTTTCCCCTTC |
| ***YAP*** | GCATGATCTGCCCTAAGGC | TGACCGCCGAGTACACCAT |
| ***Cyr61*** | AGTGGGTCTGTGACGAGGAT | GGGTTTCTTTCACAAGGCGG |
| ***FGFR2*** | AGCACCATACTGGACCAACAC | GGCAGCGAAACTTGACAGTG |
| ***Ccne1*** | GCAAACGTGACCGTTGATCC | TTGGGTAAACCCGGTCATC |
| ***Ccnd1*** | GCTGCGAAGTGGAAACCATC | CCTCCTTCTGCACACATTTGAA |
| ***β-Actin*** | GTTGAGAACCGTGTACCATGT | TTCCCACAATTTGGCAAGAGC |
